# Supplementary figures and images for: Gut microbiota metabolically mediate intestinal helminth infection in zebrafish
Source: mSystems. 2024 Aug 27;9(9):e00545-24. doi: 10.1128/msystems.00545-24 (PMC11406965; doi:10.1128/msystems.00545-24)

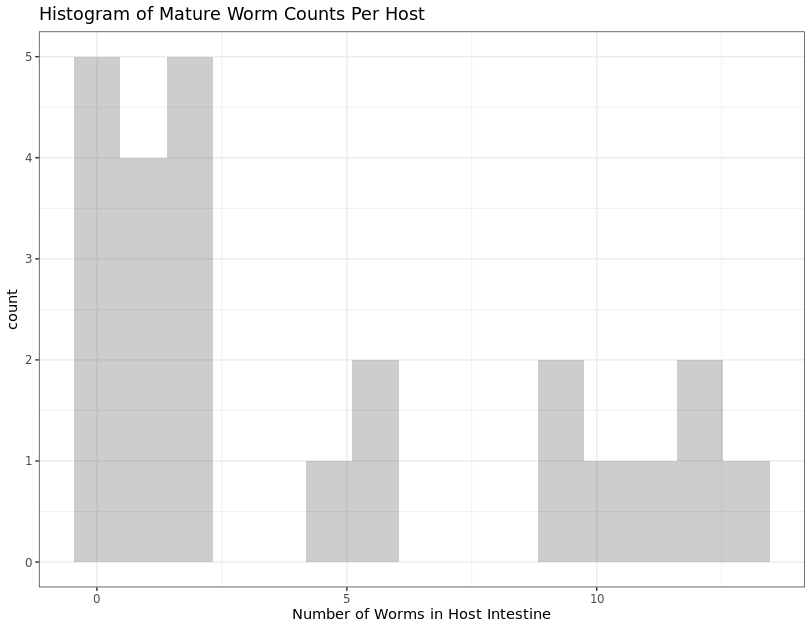

Supplement: Figure S1 — Distribution of mature Pseucapillaria tomentosa worms. [file msystems.00545-24-s0001.tiff]

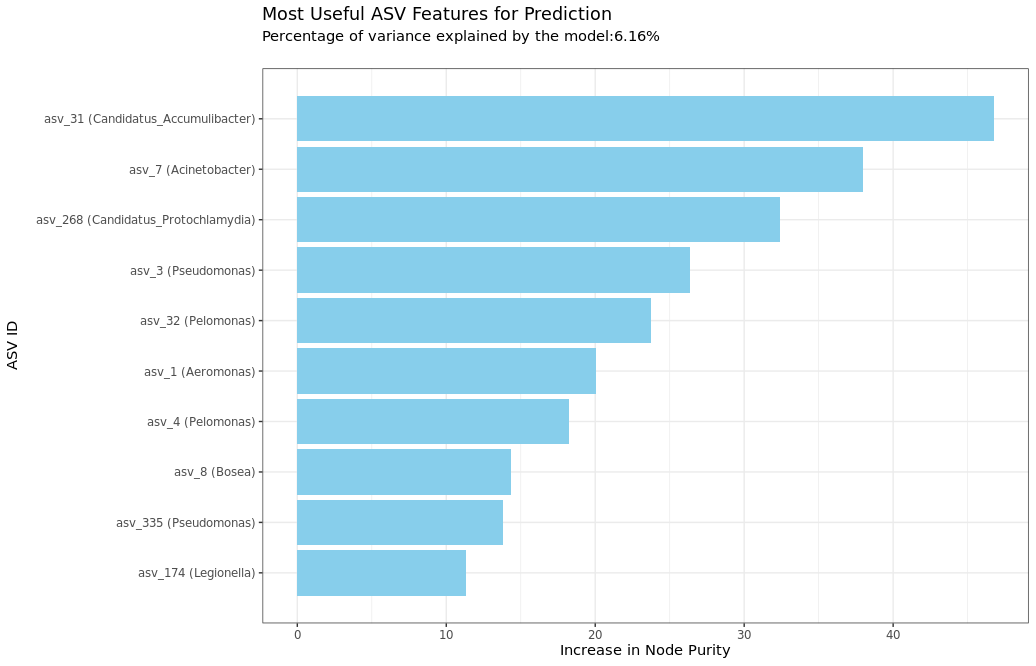

Supplement: Figure S2 — The 10 most important features based on the increase in node purity for regression of helminth worm burden. [file msystems.00545-24-s0002.tiff]
