# Supplementary material for: Gut microbiota metabolically mediate intestinal helminth infection in zebrafish
Source: mSystems. 2024 Aug 27;9(9):e00545-24. doi: 10.1128/msystems.00545-24 (PMC11406965; doi:10.1128/msystems.00545-24)
Supplement: Legends — for supplemental figures. [file msystems.00545-24-s0003.docx]

Figure S1. Distribution of mature *Pseudocapillaria tomentosa* worms quantified during dissection of intestinal tissue 29 days after initial helminth egg exposure.

Figure S2. The ten most important features based on the increase in node purity for regression of helminth worm burden measured 29dpe on microbiota relative abundances at 0dpe, just prior to parasite egg exposure.
