# Supplementary material for: Gut microbiota metabolically mediate intestinal helminth infection in zebrafish
Source: mSystems. 2024 Aug 27;9(9):e00545-24. doi: 10.1128/msystems.00545-24 (PMC11406965; doi:10.1128/msystems.00545-24)
Supplement: Supplemental Tables — Tables S1 and S2. [file msystems.00545-24-s0004.docx]

Supplementary Table 1. Coefficients table of metabolites which are linked to IHP burden (FDR<0.1) as measured 29dpe.

Supplementary Table 2. Coefficients table of PERMANOVA results testing the relationship between NAE abundance and microbiome composition prior, as well as the interaction of NAE abundance and prior antibiotic exposure at 0dpe and 29dpe.
